# Comparative Evaluation of Nasopharyngeal and Oropharyngeal Swab Based Rapid SARS-CoV-2 Antigen Detection and Real-Time RT-PCR for Diagnosis of COVID-19 in Tertiary Care Hospital

**DOI:** 10.7759/cureus.16785

**Published:** 2021-07-31

**Authors:** Arpana Singh, Pratima Gupta, Yogendra P Mathuria, Deepjyoti Kalita, Amber Prasad, Prasan K Panda, Yogesh Bahurupi, Biswajeet Sahoo, Balram Ji Omar

**Affiliations:** 1 Microbiology, All India Institute of Medical Sciences, Rishikesh, Rishikesh, IND; 2 General Medicine, All India Institute of Medical Sciences, Rishikesh, Rishikesh, IND; 3 Community and Family Medicine, All India Institute of Medical Sciences, Rishikesh, Rishikesh, IND

**Keywords:** covid 19, rapid antigen test, rt-pcr, sars cov-2, rdt

## Abstract

Purpose: To assess and compare the diagnostic accuracy of the GenBody COVID-19 Antigen kit (GenBody Inc., Cheonan, South Korea) available in the market with the gold standard reverse transcription-polymerase chain reaction (RT-PCR) assay to detect severe acute respiratory syndrome coronavirus-2 (SARS-CoV-2).

Methods: Nasopharyngeal and oropharyngeal swabs were collected from suspected coronavirus disease 2019 (COVID-19) patients and tested by RT-PCR and GenBody Rapid antigen kit. Performance characteristic of the antigen kit was calculated.

Results: We tested nasopharyngeal swabs and oropharyngeal swabs (n=240). Amongst the 102 positive RT-PCR samples, the rapid antigen test detected 36 as positive, showing an overall sensitivity of 35.3%. All the samples detected positive with the antigen rapid test were also detected positive by RT-PCR.

Conclusion: The performance of the rapid antigen kit was good with respect to high viral load samples, whereas those with lower levels were missed. Unfortunately, the overall low sensitivity of the antigen kit does not allow using it alone as the frontline testing kit for COVID-19 diagnosis.

## Introduction

The coronavirus disease 2019 (COVID-19) pandemic has created havoc worldwide since it was first recorded in Wuhan, the capital city of Hubei province, China, in December 2019 [1]. The severe acute respiratory syndrome coronavirus 2 (SARS-CoV-2) is a single-stranded RNA virus that belongs to the genus β of Family Coronaviridae. Being highly transmissible, this novel coronavirus disease (COVID-19) has spread fast worldwide [2]. The World Health Organization (WHO) declared COVID-19 as a pandemic on 11 March 2020 [3]. The gold standard tests to diagnose SARS-CoV-2 infections are viral culture and reverse transcription-polymerase chain reaction (RT-PCR) assays [4]. Although it takes a few hours for RT-PCR to detect the nucleic acid and isolation of the virus from samples takes some days, but these tests need a specialized instrument and trained people in that particular field. There is an upsurge in the number of cases, especially in countries like the United States, Brazil, and India. Therefore, rapid tests for SARS-CoV-2 might play an important role in mass screening to facilitate prevention and control of disease and screening before any surgical procedure [1].

The rapid antigen detection (RAD) tests are immunochromatographic tests, commercially available as lateral flow detect viral antigen by the immobilized SARS-CoV-2 antibody coated on the device. These lateral flow assays which use monoclonal antibodies specific to SARS-CoV-2 antigens extracted from the nasopharyngeal and oropharyngeal swabs can be utilized as the screening tests if their diagnostic accuracy were similar to that of the real-time RT-PCR assays. The RAD test takes less time and gives the results within 30 min without the need for any specialized instrument. Hence, RAD tests can drastically improve turn-around time and reduce the workload in already strained diagnostic hospitals and laboratories. As per WHO, the role of RAD tests to detect antigen for SARS-CoV-2 needs to be further evaluated and is not recommended for clinical diagnosis [5].

The purpose of this study is to assess the role of the GenBody COVID-19 Antigen test (GenBody Inc., Cheonan, South Korea) for laboratory confirmation of the cases. The objective of the study was to estimate the performance characteristics of the RAD test to detect SARS-CoV-2.

## Materials and methods

Population and study settings

COVID-19 suspected cases and their contacts were included in the study from January to March 2021 in the screening outdoors and indoor facilities at a tertiary care hospital. Respiratory samples (nasopharyngeal and oropharyngeal swabs) were collected from suspected COVID-19 cases (n=240) with the following conditions: (1) asymptomatic and symptomatic individuals with a history of close contact with confirmed cases, (2) patients with acute respiratory infections, 3) asymptomatic individuals who wanted to get themselves tested for travel, and 4) pre-operative patients. The study was approved by the Institutional Ethics Committee Review Board at All India Institute of Medical Sciences (AIIMS), Rishikesh (AIIMS/IEC/21/09)

Interventions

Performance characteristics of the SARS-CoV-2 antigen detection kit were analyzed in comparison to the RT-PCR assay. 

Clinical specimens

Respiratory specimens, nasopharyngeal swabs, and oropharyngeal swabs were collected. Samples were collected and mixed in viral transport media (VTM) and immediately transported to the virology laboratory maintaining the cold chain where these samples were tested with RT-PCR and rapid antigen test kits simultaneously. All the specimens were processed in biosafety cabinet level 2 (BSL 2 Advanced) following all infection control practices.

Viral RNA extraction

Automated system Kingfisher Flex 96 system (Thermofisher Scientific Inc., Waltham, Massachusetts, US.) was used to extract SARS-CoV-2 RNA from 200 μL of a sample. Extraction was performed according to the manufacturer’s instructions using a magnetic bead-based MagRNA extraction kit (Thermofisher Scientific Inc., Waltham, Massachusetts, US). Viral RNA extract was eluted in 80 μL of the elution buffer and an RT-PCR assay was performed.

SARS-CoV -2 RNA detection using real-time RT-PCR

Real star SARS-CoV-2 RT-PCR kit 1.0 (Altona Diagnostics GmbH, Monketsr, Hamburg, Germany), which targets *E *(Envelope) gene and *S* (Spike) gene, was used to detect SARS-CoV-2 RNA according to kit’s literature. Briefly, 10 μL of the elute was mixed with 20 μL of PCR master mix (Master A and B) to make a total of 30 μL of reaction volume. Master A and B contain all components (PCR buffer, reverse transcriptase, DNA polymerase, magnesium salt, primers, and probes) to allow reverse transcription and PCR-mediated amplification.

The CFX96 real-time thermal cycler (Bio-Rad Laboratories Inc., Hercules, CA, USA) was used for amplification. The conditions consisted of 1 cycle of 20 min at 55 °C, 2 min at 95 °C and followed by 45 cycles of 15 s at 95 °C, 45 s at 55 °C. The result was analyzed using BioRad CFX maestro qPCR analysis Software 1.1 (BioRad Laboratories, Inc., Hercules, CA, USA) in which a cycle threshold value (Ct value) < 35 for all two target genes was interpreted as a positive result.

Rapid SARS-CoV-2 antigen detection assay

Antigen test is a rapid chromatographic immunoassay for the detection of SARS-CoV-2 nucleocapsid (NP) antigen in human respiratory specimens. An aliquot of 400 μL of the sample was added to the extraction buffer given in the GenBody COVID-19 Antigen kit. The nasopharyngeal (and oropharyngeal) swab sample(s) was inserted into the extraction solution, then, the swab was mixed 8~10 times. Swabs were removed while pressing against the solution tube in order to extract most of the specimen. Four drops (~100 µL) were dropped into the sample well (S). The test results were interpreted after 15~20 min as per the kit’s instructions. Diagnostic accuracy of both tests was performed. 

Statistical analysis

Descriptive statistics were used to describe the demographic details of patients. Continuous data were presented in mean, standard deviation (SD), median, and range. Sensitivity, specificity, positive predictive value (PPV), and negative predictive value (NPV) were calculated using an online statistical tool IBM SPSS statistics for Windows software v22 (IBM Corp., Armonk, NY).

## Results

Out of the total samples tested from suspected COVID-19 cases (n=240) by real-time RT-PCR assay and rapid antigen kit, 42.5% (n = 102) were positive for SARS-CoV-2 RNA by RT-PCR, and 35.3% were positive for SARS-CoV-2 nucleocapsid antigen. The median age for COVID-19 cases was 30 years (range 5-60 years). Out of the total infected cases, 60.70% (n = 62) were males and 39.21%(n=40) were females. Symptomatic patients constitute 42.15% of total infected cases.

Real‑time RT‑PCR and SARS‑CoV‑2 antigen assays

The average cycle threshold (Ct) values in COVID-19 positive cases were 25.30 (minimum 12, maximum 33.78). The sensitivity of RAD was found to be very low compared to very high specificity, as shown in Table 1.

**Table 1:** Performance characteristics of GenBody Rapid Antigen kit RT-PCR - reverse transcription-polymerase chain reaction; PPV - positive predictive value; NPV - negative predictive value

GenBody Rapid antigen test (Index test)	RT-PCR Result (Gold standard)		
	Positive	Negative	Total
Positive	36	0	36
Negative	66	138	204
Total	102	138	240

Diagnostic Accuracy	Value (%)	95% CI
Sensitivity(%)	35.29	26.2-45.5
Specificity(%)	100	96.6-100
PPV(%)	100	87.99-100
NPV(%)	67.64	60.6-73.9

In a sub-group analysis, we divided the respiratory samples of the positive cases into two groups based on the Ct values obtained in the RT-PCR assay. This part of the study was done based on the observations by Mak et al. [5]. Based on the Ct cut-off value, samples with Ct <18.57 were designated as ‘high viral load’ while samples with Ct>18.57 were categorized as ‘normal viral load’. The corresponding Ct values, mean Ct value, Ct value range, and sensitivity were calculated as shown in Table 2. A significant difference in the performance of the rapid detection test (RDT) was found in patients with 'high viral load' and 'normal viral load' (p-value = .000022).

**Table 2:** Detection of SARS-CoV-2 by COVID-19 rapid detection kit OPS - Oropharyngeal swab, NPS - Nasopharyngeal swab, Ct - Cycle threshold value

Sample type	All samples (n=102)				
OPS+NPS	Ct value				
	Mean	Range	Tested	Positive	Sensitivity
	25.3	12.01-33.78	102	36	35.29%
Viral Load					
Ct<18.57		14.07-18.39	11	10	90.90%
Ct>18.57		18.98-30.97	90	26	28.88%

In another sub-group analysis, the test performance of RDT was calculated in different age groups as shown in Table 3. 

**Table 3:** Performance of RDT in different age groups RDT - rapid detection test

Age group (years)	Samples tested	RT-PCR positive	RDT positive	Performance of RDT (%)
<18	16	10	1	10
18-64	203	92	35	38.04
>65	21	-	-	-

## Discussion

In this study, the performance characteristics of the RAD test by the rapid antigen kit were determined to detect the SARS-CoV-2 virus in respiratory samples. Results of the RAD test were compared with the results of RT-PCR as the gold standard. We observed that the sensitivity and NPV of RAD were found to be very low compared to the RT-PCR test to identify SARS-CoV-2 infections in respiratory samples. Most of the samples having Ct values relatively on the higher side representing normal viral load were not detected by the rapid antigen kit.

In this era of the ongoing pandemic of COVID-19, prompt diagnostic testing for SARS-CoV-2 is crucial for the proper management of infected patients and to limit the spread of the virus. Molecular tests like RT-PCR assays are the standard tests for laboratory confirmation [6].

In view of an increasing number of cases every day, rapid antigen immunoassays will help to speed up the screening for the disease. Different rapid tests have been developed by companies based on SARS-CoV-2 proteins in respiratory samples. However, there are many factors that affect the analytical performances of these rapid antigenic tests like the viral load, the specimen quality, method of processing the sample, stage or timing of infection, and also the setting of the patient [1].

As reported by the manufacturer, the sensitivity and specificity of the rapid antigen test (total n = 30; positive n = 27; negative n = 3) were 90% (95% CI, 73.47-97.89%), and 98% (95% CI, 92.96-99.76%), respectively. Our results showed higher specificity (100%) but low sensitivity (35%). The batch of clinical specimens tested by the manufacturer might generally have higher viral loads (low Ct-value) than our samples.

Our test performance differs from the manufacturer’s which may be due to various factors, including the lots of kit reagents used, the quality of the sample, amount of extracted antigen, handling of samples, and their processing techniques. A negative test result does not rule out the presence of antigen. It may give negative results due to their lower levels of extracted antigen than the limit of detection of the kit. 

The lack of sensitivity of rapid diagnostic tests for virus detection is well known and similar results were already observed during the Influenza A (H1N1) pandemic [7].

A recent study by Scohy et al*.* and Ciotti et al. also observed similar overall sensitivity by performing another rapid antigen test to diagnose COVID-19 to be 30.2% and 30.77% respectively [8,9]. Other studies evaluating the performance of rapid detection tests are shown in Table 4.

**Table 4:** Performance of different rapid antigen detection tests OPS - Oropharyngeal swab, NS - Nasal swab, NPS - Nasopharyngeal swab

S.no.	Author	Place	Rapid Antigen kit	Samples	Sensitivity (%)
1	Toptan et al. (2021) [10]	Germany	Biopharm	OPS/NS	88.2-89
2	Nagura-Ikeda et al. (2020) [11]	Japan	Espline SARS-CoV-2 (FujiRebio Inc.)	Saliva	11.7
3	Chaimayo et al.( 2020) [1]	Thailand	Standard™ Q COVID-19 Ag kit (SD Biosensor®, Korea)	OPS/NPS	98.33
4	Aoki et al. (2021) [12]	Japan	Espline SARS-CoV-2 (FujiRebio Inc.)	NPS	39.7
5	Dierks et al. (2021) [13]	Germany	NADAL COVID-19 Ag kit	NPS	14.29
6	Killic et al. (2021) [14]	USA	Veritor antigen kit	NPS	66.4
7	Abdelrazik et al. (2021) [15]	Egypt	BIOCREDIT COVID 19 kit	NPS	43.1
8	Blairon et al. (2021) [16]	Belgium	GSD NovaGen SARS‐CoV‐2 (COVID‐19)	NPS	61.96
			Coronavirus Ag Rapid Test Cassette (Bio‐Rad)	NPS	62.7
			Aegle Coronavirus Ag test (LumiraDx)	NPS	64
9	Pilarowski et al. (2020) [17]	USA	Abott BinaxNOW COVID-19 Ag Card	NPS	93.3
10	Landaas et al. (2021) [18]	Norway	PanBio COVID 19 Ag (Abott)	OPS/NPS	74
11	Present study	India	GenBody Rapid Antigen kit	OPS/NPS	35.29

Low sensitivity of the rapid antigen kit may lead to false-negative results, which can be of great consequence in this pandemic. Therefore, such tests should not be recommended alone in clinical settings. Before adopting antigen-based tests we should consider the balance between cost, turnaround time, ease of performance, and more importantly its sensitivity [19]. The present study confirmed high PPV and NPV during the high viral load phase of illnesses. Hence, this may be recommended during the initial period of illness only.

## Conclusions

Given the fact that rapid antigen kits are designed such that they do not amplify the target genes like PCR, so target proteins if present in a lower amount may be missed. These rapid tests can be used for mass screening, but real-time PCR, being the gold standard, should be used to confirm the negative cases of COVID-19.
